# A bibliometric and thematic approach to agriculture 4.0

**DOI:** 10.1016/j.heliyon.2022.e09369

**Published:** 2022-05-06

**Authors:** Diego Durante Mühl, Letícia de Oliveira

**Affiliations:** a Center for Studies and Research in Agribusiness (CEPAN), Federal University of Rio Grande do Sul (UFRGS), Bento Gonçalves Avenue, 7712, Agronomy, Porto Alegre, Rio Grande do Sul, 91540-000, Brazil; b Department of Economics and International Relations (DERI), Faculty of Economics, and Interdisciplinary Center for Studies and Research in Agribusiness (CEPAN), Universidade Federal do Rio Grande do Sul (UFRGS), Rio Grande do Sul 90040-060, Brazil

**Keywords:** Agribusiness, Agricultural robots, Industry 4.0, Innovation, Social impacts

## Abstract

Researchers are developing digital solutions for agriculture. Humanity has perfected agriculture throughout history because this activity is fundamental to our existence. The agricultural sector is currently incorporating new technologies from other areas. This phenomenon is agriculture 4.0. However, a challenge to research is the integration of technologies from different knowledge fields, and this has caused theoretical and practical difficulties. Thus, our purpose with this study has been to understand the core agriculture 4.0 research themes. We have used a bibliometric analysis, and guided the data collection by the PRISMA protocol. VosViewer and Bibliometrix software generated the results. We found two main research fronts, one focussed on agriculture 4.0 development, and another on the impacts of agriculture 4.0, which may be positive or negative. We found 21 main keywords or topics researched in agriculture 4.0 related to these research fronts. These themes are within five different axes. We managed to establish a good understanding of the topics around agriculture 4.0. Future studies could focus on the responsible development of digital solutions for agriculture. This is because the social, environmental, and economic impacts of these new solutions may be positive or negative. We conclude that digital agriculture is the node technologies integration for the automation of agricultural activities.

## Highlights

• There are two main research fronts.
• Agriculture 4.0 can have both positive and negative impacts.
• Agricultural development, food supply, and climate change are high centrality and density themes.
• Crops, remote sensing, and precision agriculture are motor themes.
• Agriculture, digital agriculture, and agricultural robots are basic themes.

## 1 Introduction

Industry 4.0 is the systematic implementation of cyber–physical Systems. Computational cyberspace synchronises information from all perspectives of the production process. Thus, the machines work collaboratively in a network which makes manufacturing processes more efficient, and the supply chains more sustainable (Kumar et al., 2021; Lee et al., 2015). Factory objects gain a certain degree of autonomy, and perform intelligent actions. Thus, a paradigm shift in industrial production is taking place (Lasi et al., 2014).

This new paradigm is now extending to the agricultural sector with the possibility of creating digital agriculture emerging from 2002 onwards, with the advent of digitisation (Tang et al., 2002; Yong et al., 2002). Industry 4.0 came to the field in the form of agriculture 4.0. The new technologies are altering agriculture and transforming it into a particular new research field (Bollini et al., 2019). Thus, digital agriculture or agriculture 4.0 represents the adoption of new technologies, such as the Internet of Things, big data, cloud computing, advanced robotics, and artificial intelligence in the agribusiness production chains. The adoption of these technologies aims to increase productivity, improve efficiency, and reduce the environmental impacts of agriculture, among other benefits (Lee et al., 2015; Muhuri et al., 2019; Mukherjee et al., 2021).

The biggest digital agriculture challenge is the integration of technologies from the most diverse areas of knowledge. Overcoming the integration difficulties between technologies from different areas is fundamental for the development of agriculture 4.0 (Iglesias et al., 2020). Furthermore, innovation is not without its disadvantages. We still have to make the right decisions and guide this innovation process towards sustainability. However, to make the right decisions, we need to understand the local and global impacts of these changes. So, it is necessary to know the risks and advantages of agriculture 4.0 (Klerkx et al., 2019).

In order to understand agriculture 4.0, we need to know which themes fall within this field of study. In this sense, Klerkx et al. (2019) in a systematic literature review defined at least five different thematic axes related to digital agriculture in the social sciences: 1) introduction and adaptation of digital technologies on farms; 2) changes in farmer identity, new farmer skills and the sophistication of farm work; 3) power, ownership, privacy, and the ethics of digital agriculture; 4) agricultural knowledge and innovation; 5) economics and management of digitised agricultural production systems.

However, Silveira et al. (2021) in a systematic literature review also highlighted five other dimensions for agriculture 4.0: 1) technological; 2) economic; 3) political; 4) social; 5); environmental. Thus, the authors converge on some points but diverge on others.

Bertoglio et al. (2021) addressing the digital revolution in agriculture, presented five clusters and one theme for each cluster in bibliometric analysis. The themes found by the author were remote sensing, the Internet of Things, climate-smart agriculture, artificial intelligence, and site-specific management.

With another more technology-oriented approach, Lezoche et al. (2020) argued that there are six important areas for agriculture 4.0: 1) the big data technologies; 2) the Internet of Things; 3) knowledge model approaches; 4) artificial intelligence techniques; 5) smart agriculture; 6) precision farming techniques, and robot development.

Furthermore, other authors describing the agriculture 4.0 scenario propose a high-level technological architecture, emphasising the importance of core technologies of intelligent agricultural systems. These systems are based on: 1) sensors (perception) and robots (actuation); 2) internet of Things (protocols and connection); 3) cloud computing (data pre-processing and storage); 4) data analysis (big data and artificial intelligence techniques); 5) decision support systems (visualisation and user interface) (Araújo et al., 2021; Saiz-Rubio and Rovira-Más, 2020).

The digital agriculture and agriculture 4.0 concept deals with the new digital technologies adoption in the agricultural sector. There is still no established and unanimous concept to deal with this subject, but studies in this area have increased in recent years (da Silveira et al., 2021). In this sense, we investigate addressed topics in this new field of studies. Different literature reviews have not reached a consensus on the agriculture 4.0 thematic axes (Araújo et al., 2021; da Silveira et al., 2021; Klerkx et al., 2019; Lezoche et al., 2020; Saiz-Rubio and Rovira-Más, 2020). Thus, the purpose of the article is to understand the central themes of the agriculture 4.0 research field, using a thematic approach.

The purpose of the article is justified because there is no consensus on agriculture 4.0 between different authors. Furthermore, there is no theoretical consensus on the thematic axes of this research field. However, the adoption of emerging technologies seems to be the pillar of agriculture 4.0 (da Silveira et al., 2021). However, there is a gap related to the topics addressed in agriculture 4.0, and the thematic axes of this research field are not entirely clear. Thus, we developed an approach to fill this gap. We used a different strategy from that used in previous literature reviews. We started with a quantitative approach, emphasising the most statistically relevant themes.

Next, we developed a literature review, method, results, discussion, and study conclusions. We present in the results’ most discussed themes in this new research field. Therefore, the present study contributes to the research field by systematising knowledge with an innovative methodology, and presents the main themes related to digital agriculture. This article provides an opportunity to understand the research field and its themes.

## 2 Literature review

### 2.1 Digital agriculture and agriculture 4.0

Agriculture is a set of activities related to plant cultivation and animal care. We can obtain food, beverage, fibre, energy, medicine, and other products from agriculture. This activity appeared about 10,000 or 11,000 years ago (Mazoyer and Roudart, 2009; Skoglund et al., 2012). We need to understand the actual agricultural transformations because this activity has a direct impact on the environment and global food security.

We realise that agriculture has evolved by incorporating new technologies when we look at history (Abbo et al., 2010; Skoglund et al., 2012). From the 18th century onwards, the modernisation of agriculture made it possible to overcome the food shortages predicted by economist Thomas Malthus (Overton, 1996). In this sense, industry and agriculture are not separate sectors, but are interdependent. Innovation in one sector affects the other. When James Watt, in the 18th century, improved the steam engine, it sparked the first industrial revolution. However, this also caused a significant increase in productivity, the expansion of cities, and a consequent rural exodus. Moreover, after the Second World War, the third industrial revolution occurred with the creation of robotics and the use of computers in industrial structures. This would later give rise to the concept of Industry 4.0.

Industry 4.0 is the fusion between the virtual world and the real world in the context of smart factories. This phenomenon is possible due to cyber-physical systems. These are the result of the combination of technologies such as the Internet of Things, big data, cloud computing, advanced robotics, artificial intelligence, additive manufacturing (3D printing), hybrid manufacturing (additive and machining on the same machine), and other technologies. These technologies have spread to other areas, sectors, and businesses, such as aviation, agriculture, and healthcare (Kumar et al., 2021; Aydin and Kahraman, 2022). Nevertheless, the influences these innovations and new technologies may have are not always clear. Replacing a conventional process can bring new economic, social, and environmental impacts (Arifin et al., 2022).

Currently, researchers are discussing the application of digital technologies to agriculture (Lee et al., 2015; Muhuri et al., 2019; Tang et al., 2002; Yong et al., 2002). These technologies have great potential to promote more sustainable practices and food security. Many researchers are searching for the optimisation of the use of scarce resources in the agricultural sector, which is why this topic has gained so much attention in recent years (Bertoglio et al., 2021; Mukherjee et al., 2021). Thus, digital agriculture is a connected system that encompasses sensors, robots, software products, farmers, external consultants, data services, and cloud platforms to optimise the productive agricultural process. However, in this process, human intervention is essential in making key decisions (Esenam, 2017; Eweoya et al., 2021). Agriculture incorporates Industry 4.0 technologies, which increases productivity and efficiency; additionally, it may bring many benefits to agriculture (Mukherjee et al., 2021).

Nevertheless, many times the farmer seems to be a passive spectator of this revolution in agriculture (Bollini et al., 2019). The new technologies of Industry 4.0 can have several impacts on agriculture. These could extend to social, environmental, and economic aspects, with unknown consequences (Arifin et al., 2022). Thus, we developed a methodology to understand the themes and resolve the ambiguities of this new research field.

## 3 Materials and methods

### 3.1 Methodology design

The method consisted of a content analysis carried out from a meta-analysis. We applied bibliometrics with concrete objectives to bring contributions to the research field, given some precepts presented by Kraus et al. (2020). First, we explored the literature with quantitative methods (Haddow, 2018; Navrotsky and Patsei, 2021). In addition, the study is not limited to a mere bibliometrics count.

We adopted the PRISMA – Preferred Reporting Items for Systematic Reviews and Meta-Analyses – as a strategy for retrieving articles, as it is a well-established method for carrying out systematic reviews and meta-analyses (Galvão et al., 2015; Page et al., 2021). Thus, we developed a content analysis based on the meta-analysis of bibliometrics data. We used keyword counting and a strategy map as a starting point for reading the documents and presenting results. In other words, a quantitative method was the starting point.

To reduce the possibility of errors and ease the integration and analysis of data with different softwares, we used one database only. Thus, we chose Scopus as it is the largest database of abstracts and citations of literature that have been peer-reviewed (Elsevier, 2021).

### 3.2 Data collection

**Figure 1:**
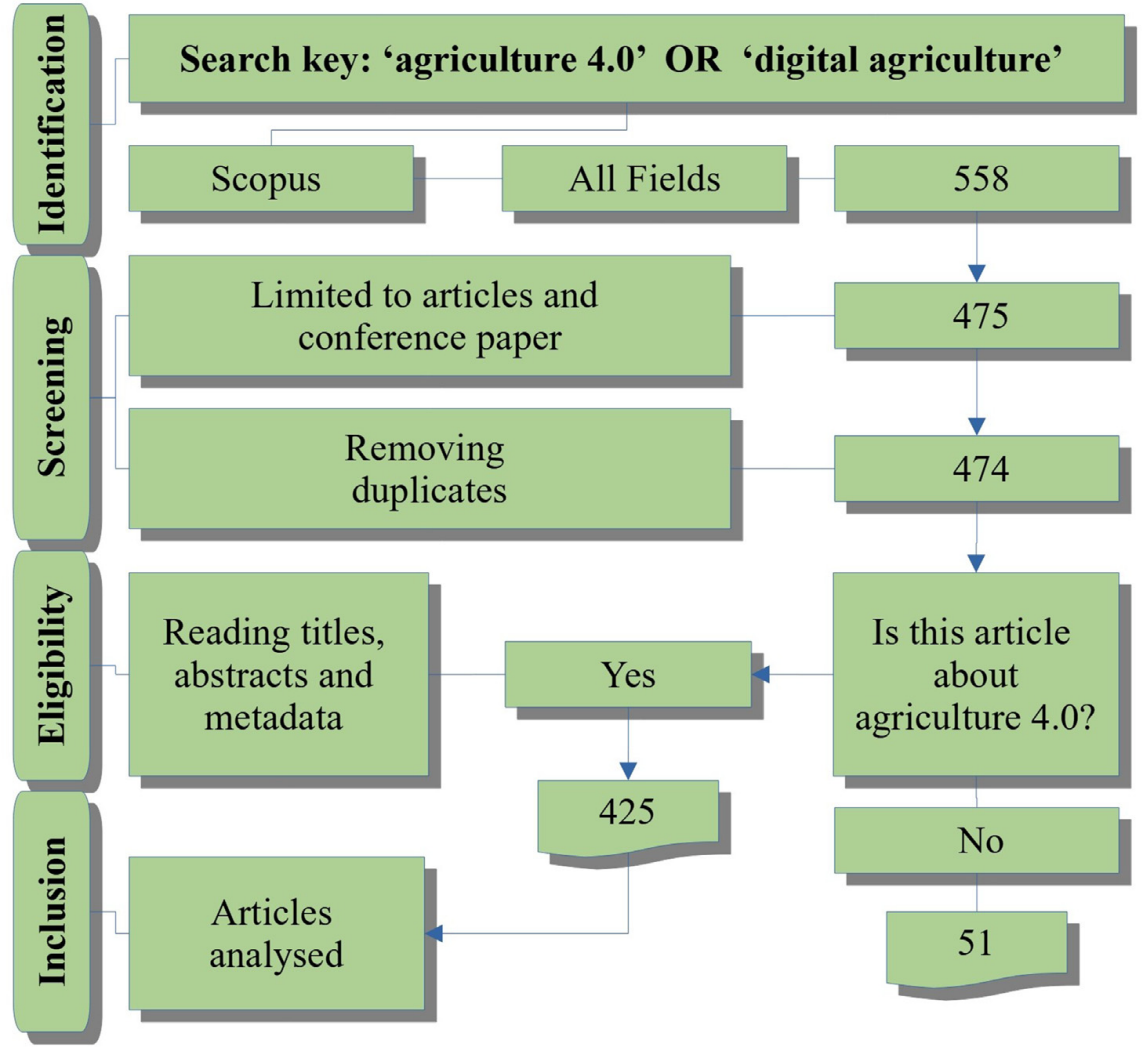
PRISMA protocol.

We based the document retrieval on the Prisma protocol (Galvão et al., 2015; Page et al., 2021) and retrieved the documents on October 13, 2021, using the search key: ‘agriculture 4.0’ or ‘digital agriculture’. The research examined the occurrence of terms in the title, abstract, and keywords of the studies. We did not establish a time frame of knowing when and where the concept first appeared. The Scopus base returned 558 documents (see Figure 1).

We limited the search to complete and conference articles in order to maintain a standard in the metadata, which facilitates the operationalisation and analysis performed with the software programs. Thus, 475 records remained. We removed one duplicate article. We read the titles, abstracts, and metadata of the articles in the screening process. Only those articles that answered the following question positively – ‘Is this article about agriculture 4.0?’ – were classified in this phase. Thus, 425 articles remained. These 425 articles were included in the analyses using the Bibliometrix and VosViewer software.

### 3.3 Data analysis

With recent developments of bibliometrics software, it is possible to analyse a scientific field, topic, or subject of interest. Software programs process bibliometric data by mathematical algorithms. This involves statistical rules, word count laws, co-citation count, bibliographic coupling, keyword co-occurrence, and other techniques (Aria and Cuccurullo, 2017; Van Eck and Waltman, 2010). We chose the Bibliometrix software package and VosViewer programs because the keyword count and strategy map creation features were adequate for this study.

The Bibliometrix^1^ package is an open-source tool programmed in the R language. This package is capable of performing a comprehensive scientific mapping. It also has several graphical and statistical features with good flexibility and frequent updates (Aria and Cuccurullo, 2017).

We used Bibliometrix features like collaborative network analysis and strategic mapping. When two or more authors had collaborated on an article, the software created a collaborative network. In this way, we were able to understand the dynamics of the production of the studies.

We used the strategy map to detect and visualise conceptual subdomains. The algorithm uses co-word and h-index indicators to create a thematic map in a two-dimensional strategy diagram. Thus, the strategy map shows a set of research themes (Aria et al., 2020; Cobo et al., 2015; Hirsch, 2005). Keyword networks form first. The keywords that appear most together form a network of words. A high-density network forms if the keywords appear together many times. Then, the software calculates the relationship that a network of words establishes with other networks of words. From there, the software groups the words according to subject areas, distributing the themes according to centrality and density. Centrality measures the degree of interaction that a keyword network establishes with other keyword networks. Density measures the internal strength of a network: that is, how closely these words relate to each other. We may classify the themes into four groups based on centrality and density (Cobo et al., 2015).

1 Motor themes: well-developed and important research field structuring themes. On the strategic map, they appear in the upper right quadrant.
2 Niche themes: very specialised and peripheral themes. Located in the upper left quadrant.
3 Emerging or declining themes: themes with low density and low centrality represent emerging or declining themes. Lower left quadrant.
4 Basic themes: important themes, but not yet well-developed in the research field. Located in the lower right quadrant, these themes are basic, general, and transversal to the research field.

VosViewer^2^ software is capable of creating networks between concepts, terms, and keywords. The VOS—the visualisation of similarity algorithm generates the VosViewer maps, and the distance between any object pair reflects their similarity with as much mathematical precision as possible (Van Eck and Waltman, 2019). We use the feature to create co-occurrence networks with keywords. The keywords used by authors form nodes. Node size indicates how often a keyword occurs. The link between nodes is proportional to the co-occurrence of the words (Van Eck and Waltman, 2014).

We present the results found below. We will revisit some aspects of the methodology to improve understanding during the presentation of the results.

## 4 Results

We performed metadata analysis of 425 documents. The R Bibliometrix package running on R Studio software with the Biblioshiny interface helped in the treatment and execution of part of the bibliometrics analyses. The sample consisted of 233 articles and 192 conference papers published in 236 journals.

### 4.1 Research field context

**Figure 2:**
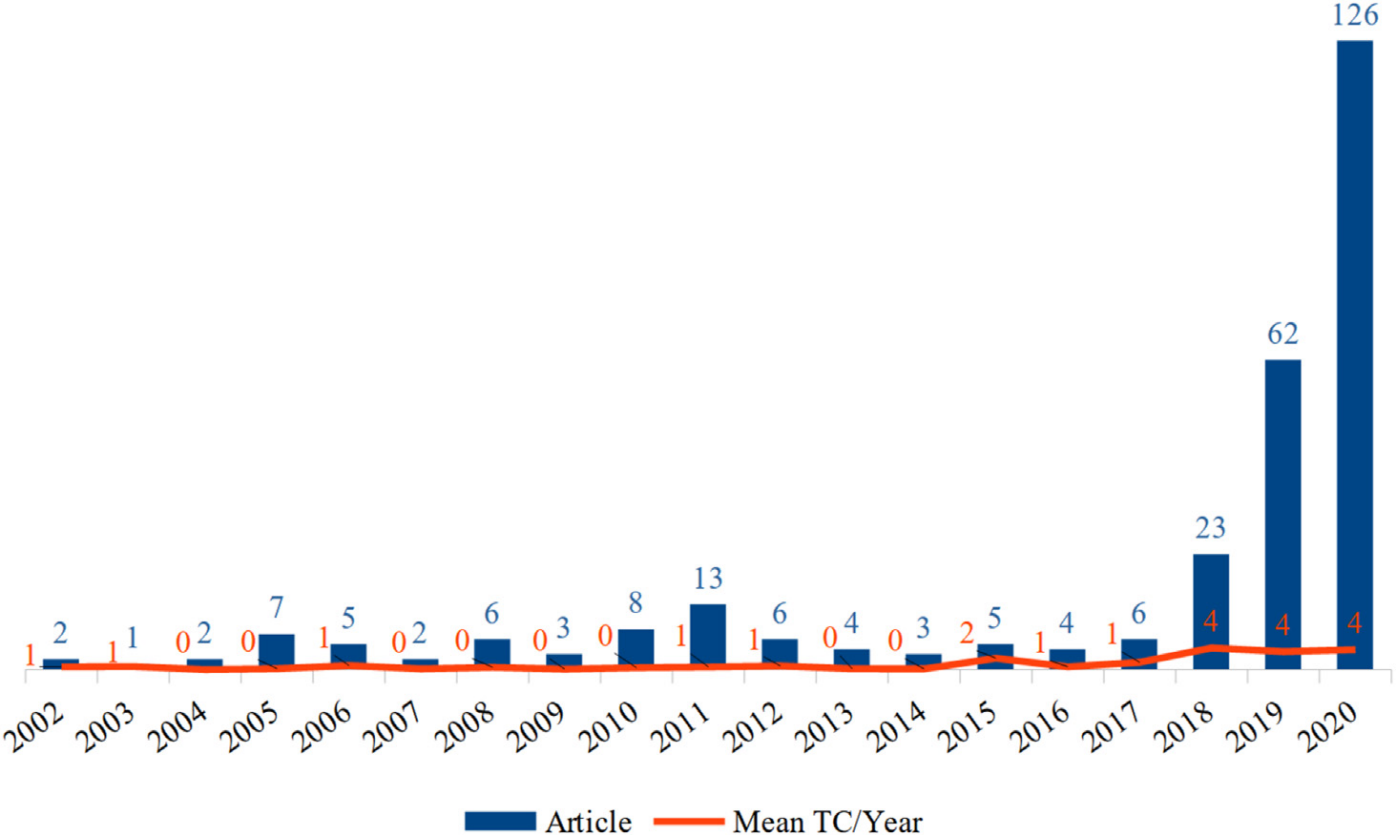
Publications growth.^3^

Figure 2 shows the growth in the number of publications over the years, and the average number of citations per year. The calculation of the average number of article citations is based on the number of years of published articles; this technique allows for time compensation and a more assertive comparison between years.

The first studies to use the concept of digital agriculture are from 2002. These addressed precision agriculture, digital earth, information agriculture, virtual agriculture, digital agriculture issues, and the viability of these systems. At that time, the main research contents for the development of digital agriculture were: the construction of a database, the creation of standards for the metadata, the development of monitoring systems, the development of forecasting systems for decision-making, and an information release system (Tang et al., 2002; Yong et al., 2002). However, there were a small number of publications on these subjects for a few years after that.

There has been a significant increase in scientific production in recent years. In 2018, publications grew by 283% compared to the previous year; in 2019, the growth was 169%; and in 2020, there was still a growth of 103%.

In 2018, there was a 197% increase in the average number of articles citations. The article ‘Agriculture 4.0: Broadening Responsible Innovation in an Era of Smart Farming’, published in 2018, has the highest number of citations (89) currently. Thus, Table 1 complements this analysis. The table's purpose is to provide a contextualisation of the authors, years, and the impact of the articles. Among the most-cited articles, the oldest is from 2015. We focus only on the three articles with the highest impact in order to start addressing the content of studies. It is a good starting point because the content of these articles provides a general and introductory overview of the research field.

**Table 1:** Articles with the greatest impact.

Author/Year	Article	TC^4^	TC/year
Rose and Chilvers (2018)	Agriculture 4.0: broadening responsible innovation in an era of smart farming	89	22.2500
Klerkx and Rose (2020)	Dealing with the game-changing technologies of agriculture 4.0: how do we manage diversity and responsibility in food system transition pathways?	68	34.0000
Rotz et al. (2019)	Automated pastures and the digital divide: how agricultural technologies are shaping labour and rural communities	65	21.6667

The most cited article in this collection investigated the impact that technologies, such as artificial intelligence, robotics, and the Internet of Things, can have on agriculture productivity and eco-efficiency. The study also points out the need to consider the social implications of the agriculture 4.0 revolution (Rose and Chilvers, 2018).

The second most cited article, with 68 citations, was “Dealing with the game-changing technologies of agriculture 4.0: how do we manage diversity and responsibility in food system transition pathways?” published in 2020. The article addressed some social consequences of the 4.0 revolution in agriculture. The authors pointed to a possible inconsistency that accompanies the process of innovation in agriculture: many authors promote agricultural innovation as a necessity for food security when, in reality, social inequalities and other factors cause the lack of access to food. We cannot guarantee food security to marginalised groups simply by increasing agricultural productivity (Klerkx and Rose, 2020).

The most cited third article addresses some of trends of North America agriculture, with special attention devoted to Canada. The authors highlighted some tensions like the increase in land and automation costs and the development of a bifurcated labour market, which is highly sophisticated on the one hand, and low skilled on the other. The authors also devoted special attention to issues related to security and digital data control (Rotz et al., 2019).

Since 2018, publications have increased substantially. Agriculture 4.0 researchers published the three most cited articles after 2018, and have a common concern: the impacts of agriculture 4.0. In summary, these impacts are in production efficiency, global food security, land costs, automation costs, and the labour market (Klerkx and Rose, 2020; Rose and Chilvers, 2018; Rotz et al., 2019).

**Figure 3:**
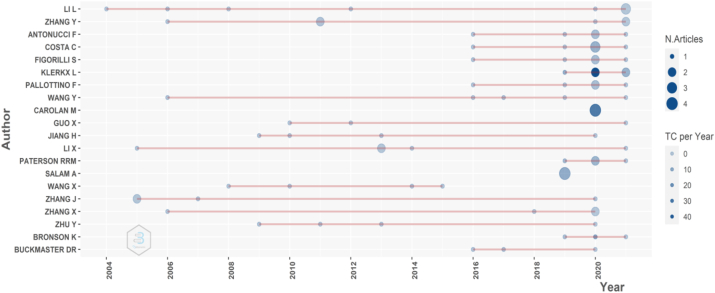
Most productive authors.

We can observe the impact of an article from the citations it receives. The academic community recognise that a highly cited work is important, but, on the other hand, some authors can contribute by the number of studies they publish. Thus, some authors tend to publish many articles, and many authors publish few articles. Typically, experts in the study field tend to publish many articles (Osareh and Mostafavi, 2011; Tran and Aytac, 2021). In the sample collected, 89% of authors published only a single article (1366 authors), and 7% of authors published two articles (113 authors). Only one author published seven articles. Figure 3 shows the most productive authors over the years to contrast and complement the previous analysis.

Li, L. published seven articles. The researcher published his first article in 2004. After that, the author also published one article each year in 2006, 2008, and 2012. Finally, in 2021, Li, L. collaborated on three publications on the subject.

In his 2004 article, Li, L. explored software architecture solutions for digital agriculture. In 2006, the author continued studying acquisition and analysis methods through the Global Positioning System (GPS). Finally, in 2008, Li, L. introduced a collection digital data device for dynamic monitoring; the device includes an infrared image sensor, an ARM and DSP-based data processing module, a GPS system, and a data link (Li et al., 2004, 2008; Yuan et al., 2006). In 2021, the author participated in publications such as “Evaluation of different deep convolutional neural networks for detection of broadleaf weed seedlings in wheat” (Zhuang et al., 2021). Therefore, the author with the most publications is an expert focussed on technological development solutions for digital agriculture.

So far, we find two research fronts: 1) researchers concerned with the impacts of agriculture 4.0 (Klerkx and Rose, 2020; Rose and Chilvers, 2018), and 2) researchers focussed on the technological development of agriculture 4.0 (Figorilli et al., 2021; Li et al., 2008; Wang et al., 2016).

**Figure 4:**
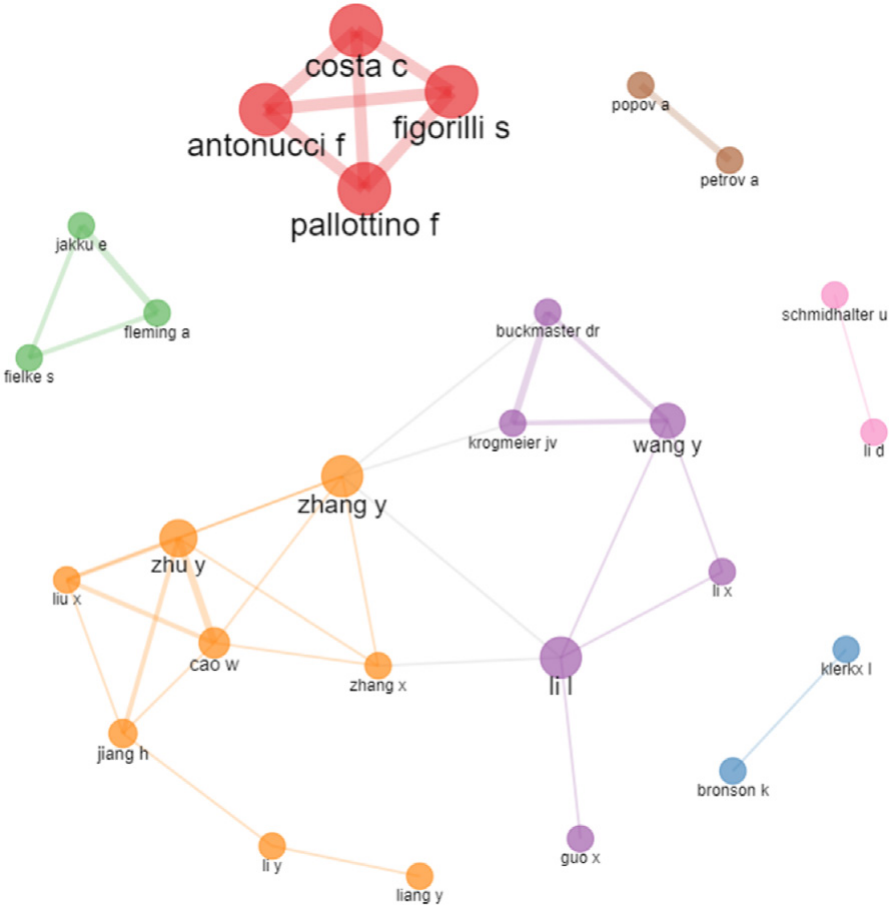
Co-authorship networks.

The authors of the two most cited articles, Rose and Klerkx, have worked on some publications together. Similarly, the two most productive authors, with the most published papers, Li, L. and Zhang Y., have jointly published. Thus, it is interesting to observe the co-authorship networks. Collaborative networks form from authors who work together to produce an article. The Figure 4 shows the authors who work in collaboration. The greater the number of publications carried out together, the thicker the line connecting two authors (Aria and Cuccurullo, 2017).

At first, we noticed that the algorithm formed some clusters of authors. Authors Li, L. and Zhang, Y., the two with the most articles published as we have seen before, appear close and establish a collaborative work network with several other researchers. Analysing the studies published by this group of authors, we concluded that these authors address issues related to remote sensing in agriculture, data collection, data processing, data analysis, and information technology for decision-making (Shen et al., 2005; Wang et al., 2016; Zhu et al., 2020). Therefore, these are researchers focussed on the technological development of agriculture 4.0.

The cluster composed by authors Costa, C. Antonucci, F. Figorilli, S. and Pallottino, F. has thick lines. These authors explore issues related to infrared vision for weed detection, devices for optimising water use in irrigation, and stereo computer vision systems for agricultural machinery (Costa et al., 2019; Figorilli et al., 2021; Pallottino et al., 2020). Thus, the authors of the two largest co-authorship networks work on solutions for digital agriculture development.

**Figure 5:**
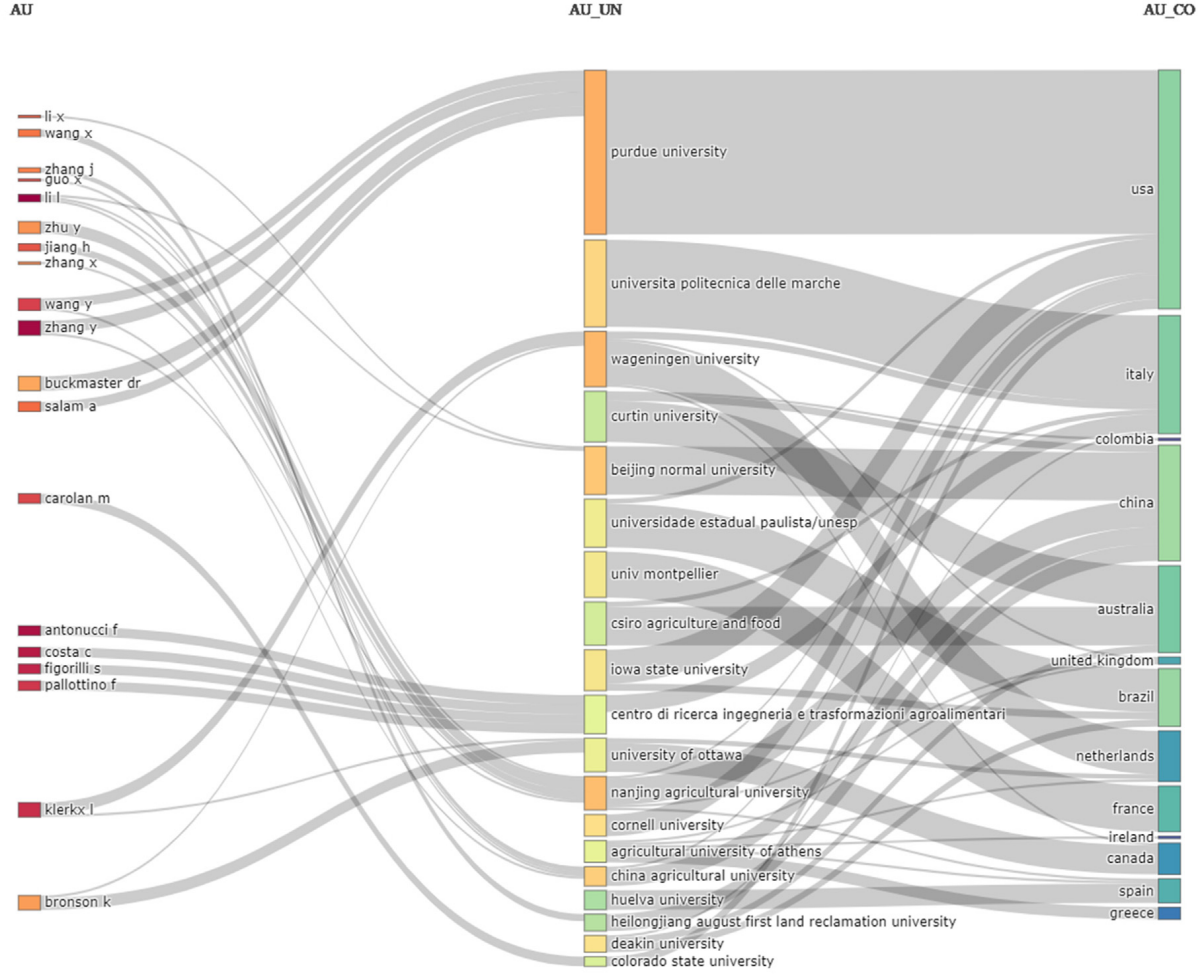
Authors, universities, and countries.

As has been observed, the authors establish collaborative networks. These may form due to research interests or because of the affiliation and countries of the authors. In this sense, in orer to situate the authors in their countries, we have included Figure 5. We continue the investigation adding elements and other points of view to understand the research field.

**Figure 6:**
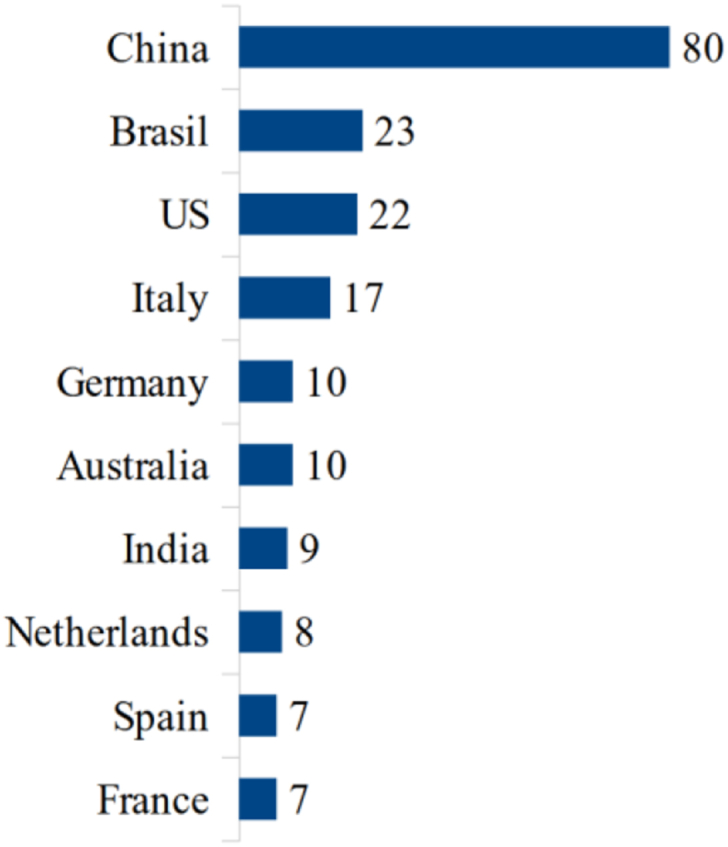
Countries with more publications.

**Figure 7:**
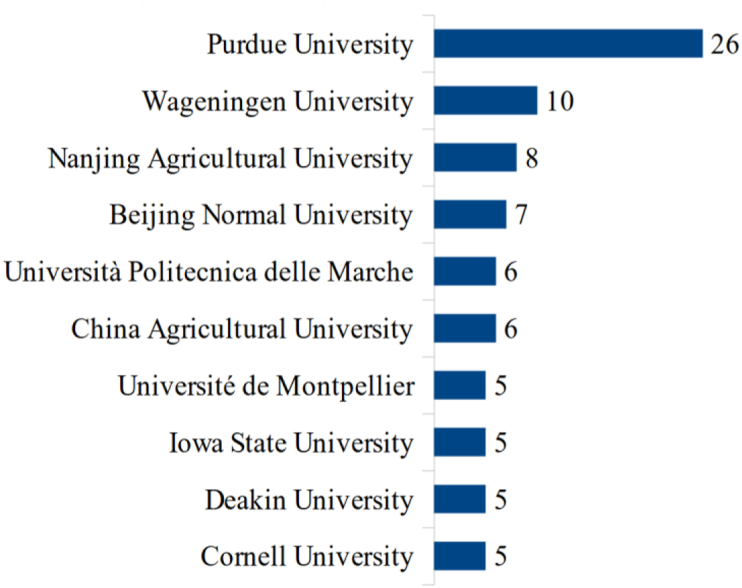
Universities with more publications.

The authors, Costa C., Antonucci F., Figorilli S., and Pallottino F., who formed a collaborative network in Figure 4 are now clearly linked by the institution where they carried out their studies. These researchers are from *Centro Di Ricerca Ingegneria and Trasformazioni Agroalimentari*, in Italy. However, there are authors linked to more than one institution like Wang Y. and Zhang Y. who are affiliated with Chinese and North American universities. This dynamic is reflected in the publications from countries and universities. At least 14 Chinese institutions, 13 US, 9 Italian and 9 Brazilian are investigating these topics (see Figures 6 and 7).

Purdue University in the US is the most published university, followed by Wageningen University in the US, but the country with the most publications is China, followed by Brazil. Thus, there seems to be a collaboration between authors of other nationalities and North American universities, reinforcing what we observed in Figure 5.

So far, we have contextualised the field of research. Over the years, we have become familiar with the publication dynamics, the works with the highest impact, the most productive authors, the collaboration of authors, the author's affiliation, universities, and countries. We also made a brief introduction to the topics researched. From this point on, we will delve deeper into issues of agriculture 4.0 with a thematic analysis.

### 4.2 Thematic analysis

We used the features of the VosViewer software to create co-occurrence networks with keywords. The keywords used by authors form nodes, node size indicates the keyword occurrence and the link between nodes is proportional to the co-occurrence of the words (Van Eck and Waltman, 2014). We considered only keywords with at least five occurrences; 142 keywords met this rule. To emphasise the main themes, the figure presents only the 20 keywords with the highest occurrence, that is, the words that most appear.

**Figure 8:**
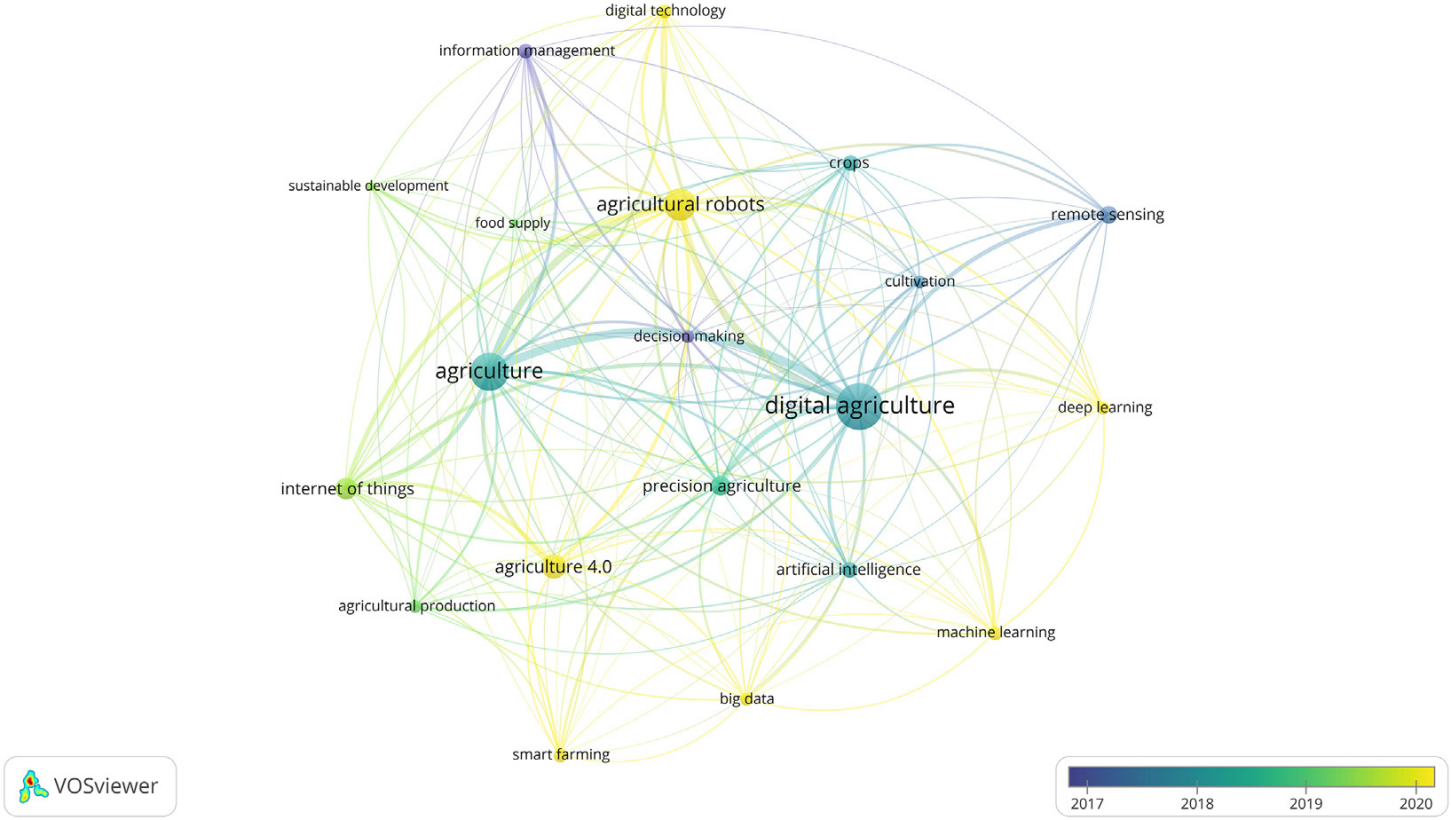
Keyword Co-occurrence networks.

As we saw earlier, most publications appear from 2018 onwards. In 2017, most studies dealt with remote sensing, information management, and decision-making, as shown in Figure 8. In 2018, the concept of digital agriculture is highlighted. From that moment on, themes such as precision agriculture and the Internet of Things gained momentum. In 2020, the theme agriculture 4.0 was highlighted, accompanied by agricultural robots, big data, smart farming, machine learning, and others.

Here it is clear that the concept of digital agriculture is older than agriculture 4.0. As we saw earlier, it appears in studies by Li L. published in 2002. The term agriculture 4.0 has only gained prominence more recently.

**Figure 9:**
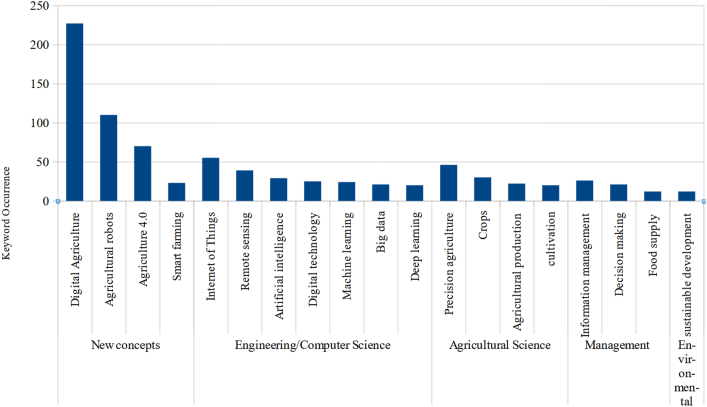
Keywords occurrence by subject area.^5^

The search engine words “digital agriculture” and agriculture form the centre of the Figure 8. Thus, it is interesting to observe the relationship between these and new words. The software calculates node size according to keyword occurrence. Figure 9 shows the research field core keywords.

The search engine words “Digital agriculture” have 227 occurrences and ‘agriculture 4.0’ has 70 occurrences. Agriculture (156 occurrences), agricultural robots (110 occurrences), the Internet of Things (55 occurrences), and the others in the Figure 9 are the research field core keywords. These are the most indexed keywords, and represent the most discussed topics in the research field of digital agriculture, or agriculture 4.0. To contextualise, we may situate each keyword on your respective subject area as showed in Figure 9.

A variety of themes form the foundation of this research field. Agricultural robots and smart farming are new concepts not directly related to another knowledge field. Thus, we consider these concepts as belonging to the new agriculture 4.0 research field. We realise many keywords are related to computer science and engineering, but agricultural and management sciences are also major knowledge areas for agriculture 4.0.

To deepen the analysis of the themes, we use a thematic map. This strategy combines performance analysis and scientific mapping tools to detect and visualise conceptual subdomains. The algorithm uses co-word and h-index analyses to create the thematic map. The algorithm classifies the themes into four groups based on centrality and density—1) motor themes: important and well-developed themes for structuring a research field; 2) niche themes: very specialised and peripheral themes; 3) emerging or declining themes: low density and centrality themes to the research field; 4) basic themes: basic, general, and transversal themes to the research field (Aria et al., 2020; Cobo et al., 2015; Hirsch, 2005).

We used the keywords plus to generate the thematic map, and set three parameters to maintain the richness of information without impairing map readability. The algorithm built the map from the 510 most frequent keywords, considering only keywords with a minimum frequency of three occurrences per 1,000 documents, with a limit of three keywords for each cluster.

The algorithm formed seven clusters dispersed in the thematic map. From the keywords and these seven thematic axes, we retrieved studies to contextualise and understand the themes in the research field.

1) Motor themes

In the upper right quadrant are the motor themes. The algorithm formed two clusters of motor themes. Crops, remote sensing, and precision agriculture form the chief cluster-driving themes. The geographic information systems, data processing, and land-use formed a secondary cluster-driving themes.

Basically, themes related to agricultural science and older technologies make up the motor themes. As we have already seen addressing the collaboration between authors, Li L. and Zhang Y. and their network of collaborators have developed research related to remote sensing in agriculture, collecting, processing and analysing data since 2005 (Shen et al., 2005; Wang et al., 2016; Zhu et al., 2020).

Currently, researchers may use satellite images to understand the behaviour of individual farmers in their climatological, logistical, and economic contexts. This type of data allows the analysis of variables that influence crop yields in real-time. Processing these data may even help to determine optimal sowing and harvesting dates based on climatic factors, or enable to making productivity previsions. Additionally, machine learning or deep learning techniques may perform this information processing (Zhang et al., 2011; 2021a; 2021b). However, remote sensing application is not limited to this.

2) Niche themes

In the upper left quadrant are the niche themes, which are peripheral and specific topics for the research field. Topics such as wireless sensor networks, neural networks, and intelligent systems are in this quadrant. In other words, we noticed a strong presence of topics related to new technologies in computer science and engineering.

The wireless sensors network is the master theme of this cluster. These sensors are the basic building block for agriculture 4.0, as they allow the connection of stand-alone devices without the need to use cables. Implementing connection is critical to the Internet of Things in the context of farms (Chehri et al., 2020; Treiber et al., 2019).

Sensors are responsible for data acquisition on farms, and networked sensors have great application possibilities in farms. Connected image sensors can analyse soil health, plant growth, or diagnose tree diseases earlier than a human can detect (Calamita et al., 2021; Hoummaidi et al., 2021). In animal care, modern sensors allow veterinarians to lead examinations on the farms with immediate tests and results (Creedon et al., 2019). Intelligent sensors and systems enable the self-diagnosis of agricultural tractors in terms of gas and fuel, thus optimising performance, thermodynamic efficiency, and the reduction of greenhouse gas emissions (Hao et al., 2018; Schlosser et al., 2020).

Like wireless network sensors, intelligent systems are niche themes. Intelligent systems can solve some more complex problems. For example, the agricultural environment has variations in temperature, humidity, light, and other parameters which affect how sensors works. However, we may train intelligent systems, neural networks, and machine learning systems to deal with this variability (Lopes et al., 2021; Taylor et al., 2020). Intelligent systems like neural networks learn, and this enables the optimisation of the operational characteristics of different agricultural equipment or systems (Batiha and Krömer, 2021; Dozono et al., 2022; Kamyshova et al., 2022).

3) Emerging or declining themes

In the lower left quadrant are emerging or declining themes. As we are approaching a field of studies that has had significant growth since 2018 onwards, the themes in this quadrant are likely emerging ones. Thus, researchers are starting to discuss these issues in the agriculture 4.0 context. The algorithm formed two clusters. Cultivation, artificial intelligence, and monitoring formed a more centralised cluster. Soils, fertilizers, and fruits formed a smaller cluster. The main emerging themes are from areas such as computer science, agricultural science, and management.

To contextualise the relationship between artificial intelligence, costs, and soil, we can think about the usage of agricultural fertilisers. Digital agriculture aims to improve the usage of fertilisers by applying the exact dosage needed by the crop (Giannoccaro et al., 2020). For this, digital agriculture checks soil parameters and water pH with sensors. So, artificial intelligence can suggest the appropriate fertilisation and water usage based on crop necessity (Hatture and Yankati, 2021).

4) Basic themes

The basic themes are shown in the lower right quadrant. These are basic, general, and transversal themes to the research field. The algorithm formed a cluster with agriculture, digital agriculture, and agricultural robot themes. We realised that digital agriculture and agricultural robots are new concepts not within other research fields that precede agriculture 4.0. Thus, these are topics not yet well developed, and which thus can be characterised as basic research themes.

Agricultural robots are still at an early stage of development, but researchers are testing these systems. Oliveira et al. (2021) identified around 62 agricultural robotic systems. The systems are relatively different, and come from different perspectives. In general, these systems can prepare the soil, carry out sowing, planting, plant treatment, harvesting, and estimation of productivity (Oliveira et al., 2021).

With the Internet of Things, there is the possibility of working with swarms of robots and small-scale unmanned aerial vehicles (drones) in collaborative systems. Drones can capture images to guide the actions of ground robots. Drones may still be used as a bridge for wireless data transfer (Shamshiri et al., 2018; Zhang et al., 2020). The Internet of Things refers to the digital interconnection of objects on a network or the Internet. This new interactive technology permeates almost every emerging and niche agriculture 4.0 topic. Overcoming incompatibilities between different devices and protocols is a challenge for the Internet of Things in digital agriculture (Iglesias et al., 2020).

Digital agriculture is not limited to the use of agricultural robots. To exemplify an Internet of Things application, we can cite a system that checks the health of animals on a farm. Cows wear collars that track various vital sign. The collar transmits the information over a wireless network. The hardware structure consists of the hub, Wi-Fi access points, and routers. The cloud system stores the data with the server-hosting database and the application server. Devices of this nature can identify abnormalities in animal health long before humans can detect them (Unold et al., 2020).

However, there are technical difficulties related to these systems. Most of today's agricultural robots are mobile with four-wheel-drive systems, but farming soil is very irregular, and this system can have several limitations. The characteristics of the agricultural environment like temperature, humidity, and dust may damage the sensors. The cameras on these devices demand high-quality images, which makes the systems more expensive. Furthermore, the computer vision algorithms of these devices still need to be optimised in order to operate with low energy consumption and high data processing (Oliveira et al., 2021).

5) High centrality and density themes

The algorithm placed agricultural development, food supply, and climate change at the very centre of the strategic map. These are the topics most discussed and related to the other clusters of the strategic map. In other words, researchers discuss these themes with emerging, basic, motor, and niche topics. These themes are more related to environmental sciences, agribusiness, and management.

Agricultural development may produce more food. Many authors justify agriculture 4.0 as a solution capable of promoting food security without harming the environment. Besides, in practice, the digitisation of agriculture may have different goals. It does not mean the same thing for different actors (Schnebelin et al., 2021). In this sense, the government has a strategic role as regulator and promoter of a fair innovation process that considers the economic, environmental, and social difficulties related to innovation in agriculture (Schnebelin et al., 2021). However, the lack of government support and incentives has been one of the main barriers to agriculture 4.0 in many countries (Kumar et al., 2021).

## 5 Discussion

We are facing a multifaceted field of research, which has grown significantly in recent years. The convergence of themes from different areas of knowledge causes some inconsistency or ambiguity in terms of agriculture 4.0 and digital agriculture, as already observed by other authors (da Silveira et al., 2021).

The day-to-day functioning of digital precision agriculture may be imprecise. In this case, agricultural activities and production chains are not optimised, and new problems arise. Digital systems can generate large volumes of erroneous data, requiring constant adjustments for sensor calibration and data interpretation. The evolution of algorithms tends to be uneven according to crop profitability. Large-scale staple cultures are well-served with innovation and technology, but other cultures must settle for much less advanced and imprecise algorithms (Visser et al., 2021).

Agriculture 4.0. may exacerbate social inequalities (Klerkx and Rose, 2020; Rose and Chilvers, 2018). In this sense, it is important to realise that there are no operations and studies related to the development of agricultural robots on the African continent (Oliveira et al., 2021). The poorest continent with the highest poverty, hunger, and lack of skilled labour is excluded from the new technological wave.

Nevertheless, there is no doubt that any innovation in the agricultural sector is welcome, especially in relation to making agriculture more sustainable. The innovation of agricultural activities is a considerable ally in the fight against climate change. Agriculture accuracy means reducing scarce resource use such as water, fertilisers, and fossil fuels. Intelligent equipment may check soil and equipment parameters, reducing the waste of essential agricultural inputs (Giannoccaro et al., 2020; Hatture and Yankati, 2021).

As of 2019, many publications address the topic of agricultural robots. These are an evolution of research and strategies initiated in 2002, which addressed the concept of digital agriculture (Tang et al., 2002; Yong et al., 2002). Several years of research and technology integration has enabled the maturation of these solutions, which are now tested and improved in factual cases (Unold et al., 2020).

However, digital agriculture is not limited to agricultural practice improvement. Agricultural robots are a disruptive technology that fundamentally change the way of doing agriculture as they almost completely replace human labour, and can be powered by renewable energy sources, like solar energy. Thus, digital agriculture is a new paradigm for the sustainability of the agricultural sector. On the other hand, we need to think about what kind of waste will be produced by the new model of agriculture, such as electronic waste, batteries, and other potential pollutants.

In this scenario, an important step is to understand the field of research itself. However, due to the multidisciplinary nature of the topic, some ambiguities arise. The difficulty of coordinating themes from different areas may reflect theoretical and practical difficulties. Taking computer vision as an example, currently there are several computer vision algorithms, but when it comes to agriculture the most suitable choice for each situation is not known (Oliveira et al., 2021).

The researchers sought to understand this new field of research and its thematic axes to resolve theoretical ambiguities. Table 2 summarises the main thematic axes according to the different authors.

**Table 2:** Thematic axes according to the different authors.

Core Themes	Klerkx et al. (2019)	Silveira et al. (2021)	Lezoche et al. (2020)	Araújo et al. (2021); Saiz-Rubio & Rovira-Más (2020).	Bertoglio et al. (2021)	Current study
1	Introduction and adaptation of digital technologies on farms	Technological	The big data technologies	Sensors (perception) and robots (actuation)	Remote sensing	Crops, remote sensing, and precision agriculture
2	Changes in farmer identity, new farmer skills and sophistication of farm work	Economic	The Internet of Things	Internet of Things (protocols and connection)	Internet of Things	Wireless sensor networks, data acquisition, and intelligent systems
3	Power, ownership, privacy, and ethics of digital agriculture	Political	Knowledge model approaches	Cloud computing (data pre-processing and storage)	Climate-smart agriculture	Artificial intelligence, monitoring, and costs
4	Agricultural knowledge and innovation	Social	Artificial intelligence techniques	Data analysis (big data and artificial intelligence techniques)	Artificial intelligence	Agriculture, digital agriculture, and agricultural robots
5	Economics and management of digitised agricultural production systems	Environmental	Smart agriculture	Decision support systems (visualisation and user interface)	Site-specific management	Agricultural development, food supply, and climate change
6			Precision farming techniques and robot development			

**Figure 10:**
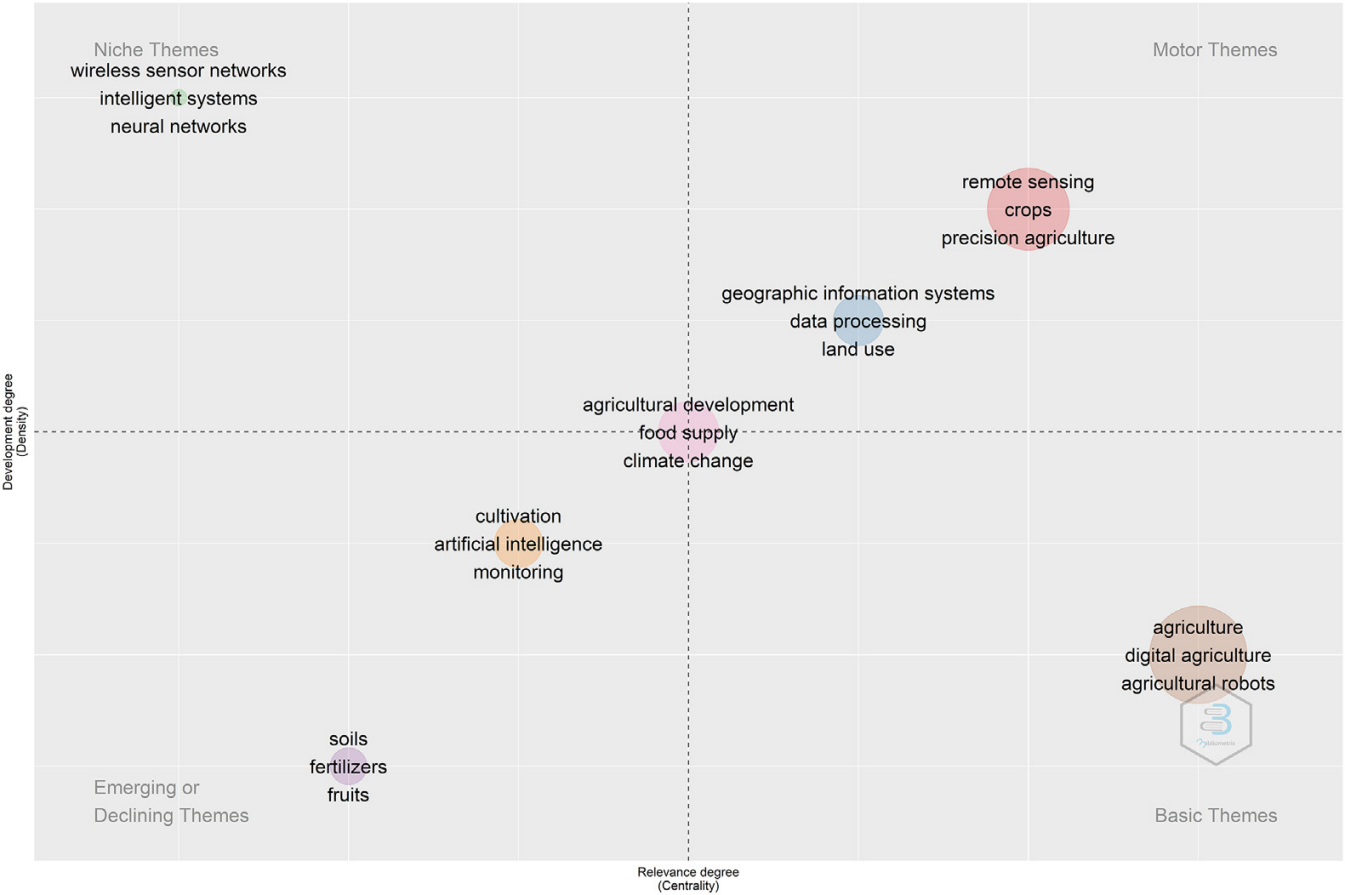
Thematic map.

This table summarises the different thematic axes according to different authors. For comparison purposes, we also added the main results of the thematic map in Figure 10. It is clear that the approach of different authors has some divergences, but there are also many similarities.

However, our approach to agriculture 4.0 allows for a more sophisticated understanding of this research field. To resolve the ambiguities, we approached the themes statistically with bibliometric techniques and consultation of the content of the articles. We may summarise the results found in the table below.

We found two main research fronts in the contextualisation of the research field. One focussed on agriculture 4.0 development, and another on the impacts of agriculture 4.0. Still, agriculture 4.0's impacts may be positive or negative. Related to these research fronts, we found 21 main keywords or topics researched in agriculture 4.0. These themes are within five different axes, as shown in Table 3.

**Table 3:** Results summary.

Research field context	Thematic analysis
Agriculture 4.0 technologies development	**1) Motor themes** a) Crops, remote sensing, and precision agriculture. b) Geographic information systems, data processing, and land-use.
	**2) Niche themes** a) Wireless sensor networks, data acquisition, and intelligent systems.
	**3) Emerging or declining themes** a) Artificial intelligence, monitoring, and costs. b) Soils, fruits, and irrigation.
	**4) Basic themes** a) Agriculture, digital agriculture, and agricultural robots.
Agriculture 4.0 impacts	**5) High centrality and density themes** a) Agricultural development, food supply, and climate change.

We found that most of the topics are related to the development of agriculture 4.0 technologies. However, the themes with greater density and centrality are focussed more on the agriculture 4.0 impacts. This is bearing in mind that themes with greater centrality and density are the thematic clusters that are most related to other thematic clusters. Thus, positive or negative current agriculture impacts are central to the research and development of agriculture 4.0 technologies.

Thus, we can hypothesise that with new technology, new positive or negative impacts arise in agriculture. Researchers and technology developers expect positive impacts, but negative impacts require new technological solutions. Thus, research in agriculture 4.0 is driven first by the need to overcome the difficulties of conventional agriculture, but second by the need to overcome the new difficulties of agriculture 4.0, itself. This dynamic characterises the technological development cycle of this research field.

The digital agriculture ramifications of this deserve attention. Most studies are focussed on technology development, but other factors may influence the acceptance of digital agriculture. Performance and effort expectation, social influence, and habits have had a significant effect in a study carried out in Nigeria about digital agriculture acceptance (Eweoya et al., 2021). Social and corporate responsibility are themes related to climate change, environmental preservation, and social impacts that researchers cannot neglect (Málovics et al., 2008).

We must consider that new technologies may have some negative impact. Research must discuss social impacts, political processes, changes in global geography themes (Klerkx et al., 2019). The related sustainability and food security discourse promote the technological development of agriculture. However, we do not know the ramifications that digital agriculture may have in the future. On the other hand, the problem of food insecurity is much more related to social injustices than to food production itself (Rose and Chilvers, 2018).

These sophisticated digital farming systems become expensive. The high investment costs can make it difficult for small and medium-sized farmers to adopt these technologies. Therefore, some researchers are considering cheap strategies for these systems. An alternative is general monitoring systems development, with actuator control, that manages different types of equipment such as water pumps, water valves, fans, among other technological resources (Zabasta et al., 2021). In addition, we need to understand the economic, social, environmental, and institutional contributions of adopting these new technologies into agricultural development. It is essential to qualify the institutions that indicate directions for the transfer of technologies, the defining of research, development, and innovation agendas (Pinto et al., 2021).

Digital agriculture is the result of integration of these diverse technologies that make up the driving, niche, and emerging themes that we have seen so far. Enthusiastically, we can say that agriculture is now “rocket science”. Autonomous machines, sensors, and satellites are beginning to gather data about soil, plants, and climate at speeds and scales unimaginable until recent times (Kirkegaard, 2019).

## 6 Conclusions

New problems arise with new solutions. The digital agriculture theme is multidisciplinary, a broad theme that involves knowledge from different areas. Approaching a field of research of this nature is challenging. In our methodological strategy, we have shown an integrated vision approach.

This study has limitations related to its themes’ diversity. The methodology always emphasises the statistically most relevant themes, but we do not represent all the themes of the research field. We use only one database, and only consider articles and conference papers. Thus with the inclusion of all documents in the research field, the results could be a little different.

Future studies could discuss and improve the methodological strategy adopted. Research may produce innovation and development in agriculture trying to avoid negative impacts, such as the environmental crisis we are experiencing caused by the irrational use of limited resources. It is no use popularising technologies with very disastrous negative impacts, we need to avoid this as much as possible.

We cannot neglect the effects of agriculture 4.0 on climate change, environmental conservation, and the social impacts caused by new technologies in agriculture. Remember that these effects can be positive or negative. New agriculture technologies' viability is related to the promise of producing more food and consuming fewer natural resources. However, aggravating social inequalities risk must be seriously addressed in different world locations. Technology can leverage the process of the concentration of power (technology, land, and capital), the marginalisation of people and, consequently, food insecurity, environment pollution, and other related aggravating factors. We need to ensure that technological development reduces and does not increase social inequalities. It is a good research topic.

The results consolidate the knowledge, and will be helpful for researchers to understand digital agriculture based on its themes. Every specialist should be familiar with the entire area of agriculture 4.0 research for a more accurate understanding. Governments and legislators must understand the need to protect people from the possible negative impacts of new technologies in the agricultural sector, especially to avoid social inequalities. Society, in general, needs to have an integrated vision of digital agriculture to promote its sustainable development.

Our article has approached the agriculture 4.0 field of studies, and presented its main themes. Digital agriculture is the integration of different technologies for the automation of agricultural activities.

## Declarations

### Author contribution statement

Diego Durante Mühl & Leticia de Oliveira: Conceived and designed the experiments; Performed the experiments; Analyzed and interpreted the data; Contributed reagents, materials, analysis tools or data; Wrote the paper.

### Funding statement

This work was supported by the 10.13039/501100002322Coordination for the Improvement of Higher Education Personnel (10.13039/501100002322CAPES), Brazil, (88887.486380/2020-00).

### Data availability statement

Data included in article/supplementary material/referenced in article.

### Declaration of interests statement

The authors declare no conflict of interest.

### Additional information

No additional information is available for this paper.

## Footnotes

1 The Bibliometrix (https://www.bibliometrix.org/index.html) software was conceived by researchers at the University of Naples Federico II in Italy.

2 VosViewer (www.vosviewer.com) is a tool developed by the Center for Science and Technology Studies at the University of Leiden in the Netherlands.

3 We do not present the result for the year 2021. We collected the data on October 13, 2021. Thus, the year was not complete. Thus, we cannot compare the year 2021 with the others. However, we considered the year 2021 for the other results.

4 Total citations.

5 These are the knowledge areas related to the most co-occurring keywords. The methodology emphasises the most statistically relevant themes, so there are other less frequent keywords and knowledge areas.

## References

[bib1] Abbo S., Lev-Yadun S., Gopher A. (2010). Agricultural origins: centers and noncenters; a near eastern reappraisal. Crit. Rev. Plant Sci..

[bib2] Araújo S.O., Peres R.S., Barata J., Lidon F., Ramalho J.C. (2021). Characterising the agriculture 4.0 landscape—emerging trends, challenges and opportunities. Agronomy.

[bib3] Aria M., Cuccurullo C. (2017). bibliometrix: an R-tool for comprehensive science mapping analysis. J. Informetr..

[bib4] Aria M., Misuraca M., Spano M. (2020). Mapping the evolution of social research and data science on 30 Years of social indicators research. Soc. Indicat. Res..

[bib5] Arifin N.A.M., Saman M.Z.M., Sharif S., Ngadiman N.H.A., Hassan M.H.A., Ahmad, Manap Z., Baharom M.Z., Johari N.H., Jamaludin U.K., Jalil M.H., Mat Sahat I., Omar M.N. (2022). Human-Cen.Tech. for a Better Tomorrow, Lectu. Notes in Mechan. Engin.

[bib6] Aydın S., Kahraman C. (2022). Aviation 4.0 revolution. Stud. Syst. Decis. Control.

[bib7] Batiha T., Krömer P. (2021). Accelerated neural intrusion detection for wireless sensor networks. Adv. Intell. Syst. Comput..

[bib8] Bertoglio R., Corbo C., Renga F.M., Matteucci M. (2021).

[bib9] Bollini L., Caccamo A., Martino C., Bozzon A., Mayo F.J.D. (2019). WEBIST 2019 - Proceedings of the 15th International Conference on Web Information Systems and Techn.

[bib10] Calamita F., Imran H.A., Vescovo L., Mekhalfi M.L., La Porta N. (2021). Early identification of root rot disease by using hyperspectral reflectance: the case of pathosystem grapevine/armillaria. Rem. Sens..

[bib11] Chehri A., Chaibi H., Saadane R., Hakem N., Wahbi M., Cristani M., J.L.C., Toro C., Zanni-Merk C., Howlett R.J., Jain L.C. (2020). Procedia Computer Science.

[bib12] Cobo M.J., Martínez M.A., Gutiérrez-Salcedo M., Fujita H., Herrera-Viedma E. (2015). 25 years at Knowledge-Based Systems: a bibliometric analysis. Knowl.-Based Syst., 25th anniversary of Knowledge-Based Systems.

[bib13] Costa C., Febbi P., Pallottino F., Cecchini M., Figorilli S., Antonucci F., Menesatti P. (2019). Stereovision system for estimating tractors and agricultural machines transit area under orchards canopy. Int. J. Agric. Biol. Eng..

[bib14] Creedon N., Robinson C., Kennedy E., Riordan A.O. (2019). Proceedings of IEEE Sensors.

[bib15] Da Silveira F., Lermen F.H., Amaral F.G. (2021). An overview of agriculture 4.0 development: systematic review of descriptions, technologies, barriers, advantages, and disadvantages. Comput. Electron. Agric..

[bib16] Dozono K., Amalathas S., Saravanan R. (2022). The impact of cloud computing and artificial intelligence in digital agriculture. Lect. Notes Netw. Syst..

[bib17] Elsevier S. (2021). https://www.elsevier.com/solutions/scopus/how-scopus-works/content?dgcid=RN_AGCM_Sourced_300005030.

[bib18] Esenam A. (2017). Overview of digital agriculture: making growers lives more productive. Int. Sugar J..

[bib19] Eweoya I., Okuboyejo S.R., Odetunmibi O.A., Odusote B.O. (2021). An empirical investigation of acceptance, adoption and the use of E-agriculture in Nigeria. Heliyon.

[bib20] Figorilli S., Pallottino F., Colle G., Spada D., Beni C., Tocci F., Vasta S., Antonucci F., Pagano M., Fedrizzi M., Costa C. (2021). An open-source low-cost device coupled with an adaptative time-lag time-series linear forecasting modeling for apple trentino (Italy) precision irrigation. Sensors.

[bib21] Galvão T.F., Pansani T. de S.A., Harrad D. (2015). Principais itens para relatar Revisões sistemáticas e Meta-análises: a recomendação PRISMA. Epidemiol. E Serviços Saúde.

[bib22] Giannoccaro N.I., Persico G., Strazzella S., Lay-Ekuakille A., Visconti P. (2020). A system for optimizing fertilizer dosing in innovative smart fertigation pipelines: modeling, construction, testing and control. Int. J. Precis. Eng. Manuf..

[bib23] Haddow G. (2018). Research Methods: Information, Systems, and Contexts.

[bib24] Hao F., Zhao X., Wang M. (2018). Intelligent agricultural machinery monitoring system based on the cloud. Adv. Intell. Syst. Comput..

[bib25] Hatture S.M., Yankati P.V. (2021). IoT-based smart farming application for sustainable agriculture. Adv. Intell. Syst. Comput..

[bib26] Hirsch J.E. (2005). An index to quantify an individual’s scientific research output. Proc. Natl. Acad. Sci. U.S.A..

[bib27] Hoummaidi L.E., Larabi A., Alam K. (2021). Using unmanned aerial systems and deep learning for agriculture mapping in Dubai. Heliyon.

[bib28] Iglesias N.C., Bulacio P., Tapia E. (2020). Proceedings of the IEEE International Conference on Industrial Technology.

[bib29] Kamyshova G., Solovyev D., Terekhova N., Kolganov D. (2022). Development of approaches to the intellectualization of irrigation control systems. Smart Innov. Syst. Technol..

[bib30] Kirkegaard J.A. (2019). Incremental transformation: success from farming system synergy. Outlook Agric..

[bib31] Klerkx L., Jakku E., Labarthe P. (2019). A review of social science on digital agriculture, smart farming and agriculture 4.0: new contributions and a future research agenda. NJAS - Wageningen J. Life Sci..

[bib32] Klerkx L., Rose D. (2020). Dealing with the game-changing technologies of Agriculture 4.0: how do we manage diversity and responsibility in food system transition pathways?. Global Food Secur..

[bib33] Kraus S., Breier M., Dasí-Rodríguez S. (2020). The art of crafting a systematic literature review in entrepreneurship research. Int. Enterpren. Manag. J..

[bib34] Kumar S., Raut R.D., Nayal K., Kraus S., Yadav V.S., Narkhede B.E. (2021). To identify industry 4.0 and circular economy adoption barriers in the agriculture supply chain by using ISM-ANP. J. Clean. Prod..

[bib35] Lasi H., Fettke P., Kemper H.-G., Feld T., Hoffmann M. (2014). Industry 4.0. Bus. Inf. Syst. Eng..

[bib36] Lee J., Bagheri B., Kao H.-A. (2015). A cyber-physical Systems architecture for Industry 4.0-based manufacturing systems. Manuf. Lett..

[bib37] Lezoche M., Hernandez J.E., Alemany Díaz M. del M.E., Panetto H., Kacprzyk J. (2020). Agri-food 4.0: a survey of the supply chains and technologies for the future agriculture. Comput. Ind..

[bib38] Li L., Liu S.-H., Wang P.-J. (2004). International Geoscience and Remote Sensing Symposium (IGARSS).

[bib39] Li L., Shen M.-X., Guo C.-X. (2008). 2008 3rd IEEE Conference on Industrial Electronics and Applications, ICIEA 2008. Singapore.

[bib40] Lopes J.V.B., Villa-Parra A.C., Bastos-Filho T. (2021). A cyber-physical system for low-cost monitoring and sensing of rural areas using sensors, microcontrollers and lora network: agriculture 4.0. Adv. Intell. Syst. Comput..

[bib41] Málovics G., Csigéné N.N., Kraus S. (2008). The role of corporate social responsibility in strong sustainability. J. Socio-Econ..

[bib42] Mazoyer M., Roudart L. (2009). https://docs.fct.unesp.br/docentes/geo/bernardo/BIBLIOGRAFIA%20DISCIPLINAS%20POS-GRADUACAO/HISTORIA%20DA%20AGRICULTURA/Historia_das_agriculturas.pdf.

[bib43] Muhuri P.K., Shukla A.K., Abraham A. (2019). Industry 4.0: a bibliometric analysis and detailed overview. Eng. Appl. Artif. Intell..

[bib44] Mukherjee S., Baral M.M., Chittipaka V., Srivastava S.C., Pal S.K. (2021). Discussing the impact of industry 4.0 in agriculture supply chain. Lect. Notes Mech. Eng..

[bib45] Navrotsky Y., Patsei N. (2021). Presented at the 2021 IEEE Open Conference of Electrical, Electronic and Information Sciences, eStream 2021 - Proceedings.

[bib46] Oliveira L.F.P., Moreira A.P., Silva M.F. (2021). Advances in agriculture robotics: a state-of-the-art review and challenges ahead. Robotics.

[bib47] Osareh F., Mostafavi E. (2011). Lotka’s law and authorship distribution in computer science using web of science (WoS) during 1986–2009. Scientometr. Inf. Manag..

[bib48] Overton M. (1996).

[bib49] Page M.J., McKenzie J.E., Bossuyt P.M., Boutron I., Hoffmann T.C., Mulrow C.D., Shamseer L., Tetzlaff J.M., Akl E.A., Brennan S.E., Chou R., Glanville J., Grimshaw J.M., Hróbjartsson A., Lalu M.M., Li T., Loder E.W., Mayo-Wilson E., McDonald S., McGuinness L.A., Stewart L.A., Thomas J., Tricco A.C., Welch V.A., Whiting P., Moher D. (2021). The PRISMA 2020 statement: an updated guideline for reporting systematic reviews. BMJ.

[bib50] Pallottino F., Pane C., Figorilli S., Pentangelo A., Antonucci F., Costa C. (2020). Greenhouse application of light-drone imaging technology for assessing weeds severity occurring on baby-leaf red lettuce beds approaching fresh-cutting. Span. J. Agric. Res..

[bib51] Pinto D.M., de Oliveira P., Minitti A.F., Mendes A.M., Vilela G.F., Castro G.S.A., Júnior L.R.N., Bogiani J.C., Rocha J.D., Novaes R.M.L., de Barros I., Rodrigues G.S. (2021). Impact assessment of information and communication technologies in agriculture: application of the ambitec-TICs method. J. Technol. Manag. Innovat..

[bib52] Rose D.C., Chilvers J. (2018). Agriculture 4.0: broadening responsible innovation in an Era of smart farming. Front. Sustain. Food Syst..

[bib53] Rotz S., Gravely E., Mosby I., Duncan E., Finnis E., Horgan M., LeBlanc J., Martin R., Neufeld H.T., Nixon A., Pant L., Shalla V., Fraser E. (2019). Automated pastures and the digital divide: how agricultural technologies are shaping labour and rural communities. J. Rural Stud..

[bib54] Saiz-Rubio V., Rovira-Más F. (2020). From smart farming towards agriculture 5.0: a review on crop data management. Agronomy.

[bib55] Schlosser J.F., de Farias M.S., Bertollo G.M., Russini A., Herzog D., Casali L. (2020). Agricultural tractor engines from the perspective of Agriculture 4.0. Rev. Cienc. Agron..

[bib56] Schnebelin É., Labarthe P., Touzard J.-M. (2021). How digitalisation interacts with ecologisation? Perspectives from actors of the French agricultural innovation system. J. Rural Stud..

[bib57] Shamshiri R.R., Weltzien C., Hameed I.A., Yule I.J., Grift T.E., Balasundram S.K., Pitonakova L., Ahmad D., Chowdhary G. (2018). Research and development in agricultural robotics: a perspective of digital farming. Int. J. Agric. Biol. Eng..

[bib58] Shen S.-L., Liu S.-H., Huang M.-F., Li X. (2005). International Geoscience and Remote Sensing Symposium (IGARSS). Seoul.

[bib59] Silveira F., Lermen F.H., Amaral F.G. (2021). An overview of agriculture 4.0 development: systematic review of descriptions, technologies, barriers, advantages, and disadvantages. Comput. Electron. Agric..

[bib60] Skoglund P., Malmström H., Raghavan M., Storå J., Hall P., Willerslev E., Gilbert M.T.P., Götherström A., Jakobsson M. (2012). Origins and genetic legacy of neolithic farmers and hunter-gatherers in Europe. Science.

[bib61] Tang S., Zhu Q., Zhou X., Liu S., Wu M. (2002). International Geoscience and Remote Sensing Symposium (IGARSS). Toronto, Ont..

[bib62] Taylor G.A., Parra C., Carrillo H., Mouazen A. (2020). 2020 IEEE 17th India Council International Conference, INDICON 2020.

[bib63] Tran C.Y., Aytac S. (2021). Scientific productivity, Lotka’s law, and STEM librarianship. Sci. Technol. Libr..

[bib64] Treiber M., Höhendinger M., Rupp H., Schlereth N., Bauerdick J., Bernhardt H. (2019). 2019 ASABE Annual International Meeting.

[bib65] Unold O., Nikodem M., Piasecki M., Szyc K., Maciejewski H., Bawiec M., Dobrowolski P., Zdunek M. (2020). IoT-based cow health monitoring system. Lect. Notes Comput. Sci. Subser. Lect. Notes Artif. Intell. Lect. Notes Bioinforma.

[bib66] Van Eck N.J., Waltman L. (2019). https://www.vosviewer.com/documentation/Manual_VOSviewer_1.6.13.pdf.

[bib67] Van Eck N.J., Waltman L., Ding Y., Rousseau R., Wolfram D. (2014). Measuring Scholarly Impact.

[bib68] Van Eck N.J., Waltman L. (2010). Software survey: VOSviewer, a computer program for bibliometric mapping. Scientometrics.

[bib69] Visser O., Sippel S.R., Thiemann L. (2021). Imprecision farming? Examining the (in)accuracy and risks of digital agriculture. J. Rural Stud..

[bib70] Wang Y., Balmos A.D., Layton A.W., Noel S., Krogmeier J.V., Buckmaster D.R., Ault A.C. (2016). 2016 American Society of Agricultural and Biological Engineers Annual International Meeting, ASABE 2016.

[bib71] Yong L., Xiushan L., Degui Z., Fu L. (2002). The main content, technical support and enforcement strategy of digital agriculture. Geo Spatial Inf. Sci..

[bib72] Yuan Y., Zhang X., Zhang Y., Li L. (2006). Digitization of grain yield and distribution information in farmland. Nongye Gongcheng XuebaoTransactions Chin. Soc. Agric. Eng..

[bib73] Zabasta A., Avotins A., Porins R., Apse-Apsitis P., Bicans J., Korabicka D. (2021). 2021 10th Mediterranean Conference on Embedded Computing, MECO 2021.

[bib74] Zhang A., Heath R., McRobert K., Llewellyn R., Sanderson J., Wiseman L., Rainbow R. (2021). Who will benefit from big data? Farmers’ perspective on willingness to share farm data. J. Rural Stud..

[bib75] Zhang D.-Y., Liu R.-Y., Song X.-Y., Xu X.-G., Huang W.-J., Zhu D.-Z., Wang J.-H. (2011). A field-based pushbroom imaging spectrometer for estimating chlorophyll content of maize. Guang Pu Xue Yu Guang Pu Fen XiSpectroscopy Spectr. Anal..

[bib76] Zhang M., Abrahao G., Cohn A., Campolo J., Thompson S. (2021). A MODIS-based scalable remote sensing method to estimate sowing and harvest dates of soybean crops in Mato Grosso, Brazil. Heliyon.

[bib77] Zhang Y., Arakawa T., Krogmeier J.V., Anderson C.R., Love D.J., Buckmaster D.R. (2020). IEEE International Conference on Communications.

[bib78] Zhu Y., Tang L., Liu L., Liu B., Zhang X., Qiu X., Tian Y., Cao W. (2020). Research progress on the crop growth model CropGrow [作物生长模型 (CropGrow) 研究进展]. Sci. Agric. Sin..

[bib79] Zhuang J., Li X., Bagavathiannan M., Jin X., Yang J., Meng W., Li T., Li L., Wang Y., Chen Y., Yu J. (2021). Evaluation of different deep convolutional neural networks for detection of broadleaf weed seedlings in wheat. Pest Manag. Sci..

